# 3D-Printed Patient-Specific Implants in Maxillofacial Reconstruction: A Systematic Review and Meta-Analysis of Clinical Outcomes and Workflow Efficiency

**DOI:** 10.7759/cureus.98224

**Published:** 2025-12-01

**Authors:** Eram Yousif, Malaz Abdalla, Hala Jumaa, Shahd Fadlelmola, Omnia Haboura, Alaa Abdelrahim, Abdelhadi A Elsayed, Mohamed A Abdelrahim

**Affiliations:** 1 Anatomical Sciences, St. George's University School of Medicine, St. George's, GRD; 2 Oral Medicine, Faculty of Dentistry, University of Khartoum, Khartoum, SDN; 3 Oral Pathology, Faculty of Dentistry, University of Khartoum, Khartoum, SDN; 4 Pathology and Laboratory Medicine, St. George's University School of Medicine, St. George's, GRD; 5 Microbiology, St. George's University School of Medicine, St. George's, GRD

**Keywords:** 3d printing, cad/cam, implant integration, maxillofacial reconstruction, patient-specific implants

## Abstract

Maxillofacial reconstruction presents unique surgical challenges. Traditional reconstructive techniques often lack precision and long-term reliability. The integration of patient-specific implants (PSIs) offers an enhanced anatomical fit and surgical efficiency. This study aimed to assess the use of 3D-printed PSIs in maxillofacial reconstruction. This study was conducted following the PRISMA 2020 guidelines, and the protocol was made available on PROSPERO. A comprehensive search was conducted in PubMed, Ovid, CINAHL, WHO Virtual Health Library, and Google Scholar using keywords related to maxillofacial reconstruction and patient-specific 3D-printed implants. The quality of the eligible studies was assessed using the Joanna Briggs Institute Critical Appraisal Tool. A meta-analysis of implant integration outcomes was conducted using the Der Simonian-Laird random-effects model. Eight studies involving 183 patients were included in the review, with six studies contributing to the meta-analysis. The pooled implant success rate was 94.7% (95% CI: 91.4% to 98.0%), indicating excellent clinical outcomes with minimal heterogeneity (I² = 0%). Most studies employed virtual surgical planning and in-house 3D printing, which reduced the production time and costs. The reported complications were minor (e.g., seroma and infection), with no major implant failures. 3D-printed PSIs demonstrate high rates of clinical success, workflow efficiency, and radiographic accuracy in maxillofacial reconstruction procedures.

## Introduction and background

The maxillofacial region is one of the most demanding and challenging areas for rehabilitation and reconstruction after traumatic accidents, tumor excision, or congenital anomalies, and it carries considerable surgical difficulty that can be attributed to the anatomical complexity of the region and functional requirements of the craniofacial skeleton [1]. Old reconstruction methods, such as autologous bone grafting and intraoperatively shaped titanium mesh, frequently lack precision and long-term efficacy [2]. These limitations have initiated change towards patient-specific implants (PSIs) fabricated by computer-aided design/computer-aided manufacturing (CAD/CAM) and three-dimensional (3D) printing technologies [1,2]. The integration of 3D printing in maxillofacial surgery facilitates the creation of implants customized to the unique anatomy of each patient, enhancing cosmetic results, surgical accuracy, and functional rehabilitation. Recent efforts to consolidate and optimize these operations include the establishment of internal PSI production pipelines, which reduce turnaround times and diminish dependence on other suppliers. It was determined that the in-house fabrication of PSIs for mandibular reconstruction significantly reduced production time (median: 4 days) and resulted in good implant retention with minimal complication rates, thereby affirming both clinical and logistical viability [3,4]. 

An essential element of this workflow is accuracy assurance, as the therapeutic efficacy of PSIs depends on exact anatomical alignment and consistent intraoperative adjustment. A thorough assessment of quality assurance methods for 3D-printed anatomical models emphasized the necessity for strong validation tools at every stage, from segmentation to print accuracy, to reduce discrepancies and enhance surgical fit [5].

Moellmann et al. (2022) conducted a retrospective evaluation of the fitting accuracy of cranial PSIs and found that 95% of the implants adhered to clinical tolerance thresholds, with deviations generally less than 1.5 mm, data that supports the reliability of CAD/CAM-based PSI workflows in cranial reconstruction [6].

The continuous enhancement of CAD/CAM design methods has significantly contributed to the broadening of PSIs. Previous research indicates that developments in digital design have facilitated the incorporation of biomechanical factors, fixation design, and topological optimization into PSI production, hence improving load-bearing capacity and long-term osseointegration [1]. The enhancements extend beyond cranial applications; Du et al. (2023) introduced a systematic in-house methodology for craniomaxillofacial PSI manufacture, highlighting reproducibility, cost-effectiveness, and efficient interdisciplinary collaboration among surgeons, engineers, and radiologists [2]. 

Notwithstanding the potential of these technologies, a recent systematic review (2023) highlighted several challenges that continue to afflict PSI-based maxillofacial rehabilitation, including discrepancies in material selection (e.g., titanium versus PEEK), surgical learning curves, and the absence of long-term comparative outcome data. Furthermore, apprehensions persist regarding regulatory supervision, cost-efficiency, and equitable access, especially in healthcare environments with limited resources [7].

This systematic review and meta-analysis aim to rigorously assess clinical performance, accuracy, workflow efficiency and cost, and the complication profile of 3D-printed PSIs in maxillofacial reconstruction. The study seeks to delineate the present capabilities and constraints of this developing reconstructive method by combining existing quantitative and qualitative evidence, while pinpointing critical areas for future research and standardization.

## Review

Materials & methods

Search Approach and Studies' Inclusion Criteria

This systematic review was done following the established guidelines of the Preferred Reporting Items for Systematic Reviews and Meta-Analyses (PRISMA) statement [8]. The protocol for this systematic review is available to the public through the link https://www.crd.york.ac.uk/PROSPERO/view/CRD420251009959. To include all relevant research published, we did an extensive electronic literature search including PubMed, Ovid, CINAHL, and the World Health Organization Virtual Health Library Regional Portal (WHO VHL). The search strategy did not set any restrictions regarding geographical location or publication date; however, we exclusively included articles in English to include the broadest possible spectrum of relevant studies.

The following search keywords were used to build the search: (“maxillofacial reconstruction” OR “facial reconstruction”) AND (“3-D Printing” OR “3D printing” OR “custom-made implant” OR “patient-specific implant”).

Additionally, the reference lists of the articles have been screened to ensure that no relevant studies were missed. All publications were imported into Zotero software for preliminary screening and to eliminate duplicates.

Inclusion and Exclusion Criteria

Studies were included if they met the following criteria, structured according to the PICOS framework: (Population) patients undergoing reconstruction for trauma, congenital defects, or oncologic resections, with no restrictions on age, gender, or ethnicity. (Interventions) include 3D-printed or customized implants using materials like titanium or PEEK and techniques such as CAD/CAM. (Comparators) are traditional methods like autografts, allografts, or standard implants. (Outcomes) of interest include implant survival, functional/aesthetic outcomes, complications (e.g., infection, implant failure), and cost-effectiveness. Studies must be conducted in clinical settings (e.g., hospitals, maxillofacial centers) and report a minimum follow-up of six months. Eligible study designs are randomized controlled trials (RCTs), non-RCTs, cohort studies, case-control studies, and case series (≥10 patients). Only peer-reviewed articles published in English will be included, along with relevant grey literature. Exclusion criteria include non-maxillofacial reconstruction, non-customized implants, case reports (n < 10), editorials, opinions, animal or in vitro studies.

The selection process was conducted in two phases. First, two independent reviewers (SHF, HJ) examined the titles and abstracts of all identified articles to select potentially relevant studies. Those deemed relevant then proceeded to a full-text assessment to ascertain their final eligibility according to the established inclusion criteria.

The included studies were mainly observational, as there were no RCTs. We mainly included (cohort studies and more than 10 case series). Out of the eight studies included in this systematic review, six provided quantitative data on implant success, defined as the number of implants placed and the number retained without failure over the follow-up period. These studies were eligible for inclusion in a random-effects meta-analysis. Additionally, we excluded case reports (n < 10), editorials, opinions, narrative reviews, animal studies, and in vitro studies, as well as non-customized implants.

Quality Assessment and Data Extraction

The quality of the studies included was evaluated for possible bias using the Joanna Briggs Institute critical appraisal checklists (https://jbi.global/critical-appraisal-tools) [9]. This tool facilitated the evaluation of the risk of bias associated with study design, implementation, and data analysis. This was found to be low to moderate. For each included study, the following data were extracted using a standardized form. Extracted variables included first author, year of publication, country, study design, sample size, underlying pathology, type of reconstructive method, 3D printing technology, radiographic outcomes, accuracy measures, and graft success rate. As well as the complication rate. Conclusions regarding feasibility, accuracy, cost-effectiveness, and 3D printing in maxillofacial reconstruction were documented to support qualitative synthesis and meta-analysis.

Statistical Analysis

The primary quantitative outcome was the proportion of successful implant cases. A meta-analysis was conducted using Jamovi (2.4.14) software, utilizing a random-effects model to estimate the overall success proportion of implants. The analysis included six studies, with effect sizes input as proportions. The DerSimonian-Laird estimator was used to calculate between-study variance (τ²).

Assessment of Heterogeneity and Publication Bias

Heterogeneity was evaluated using the I² statistic and Cochran’s Q test. Funnel plot asymmetry was assessed using Egger’s regression test and Begg’s rank correlation test. The Fail-Safe N test (Rosenthal’s method) was also applied to assess the robustness of the findings against unpublished null results.

Results

**Figure 1:**
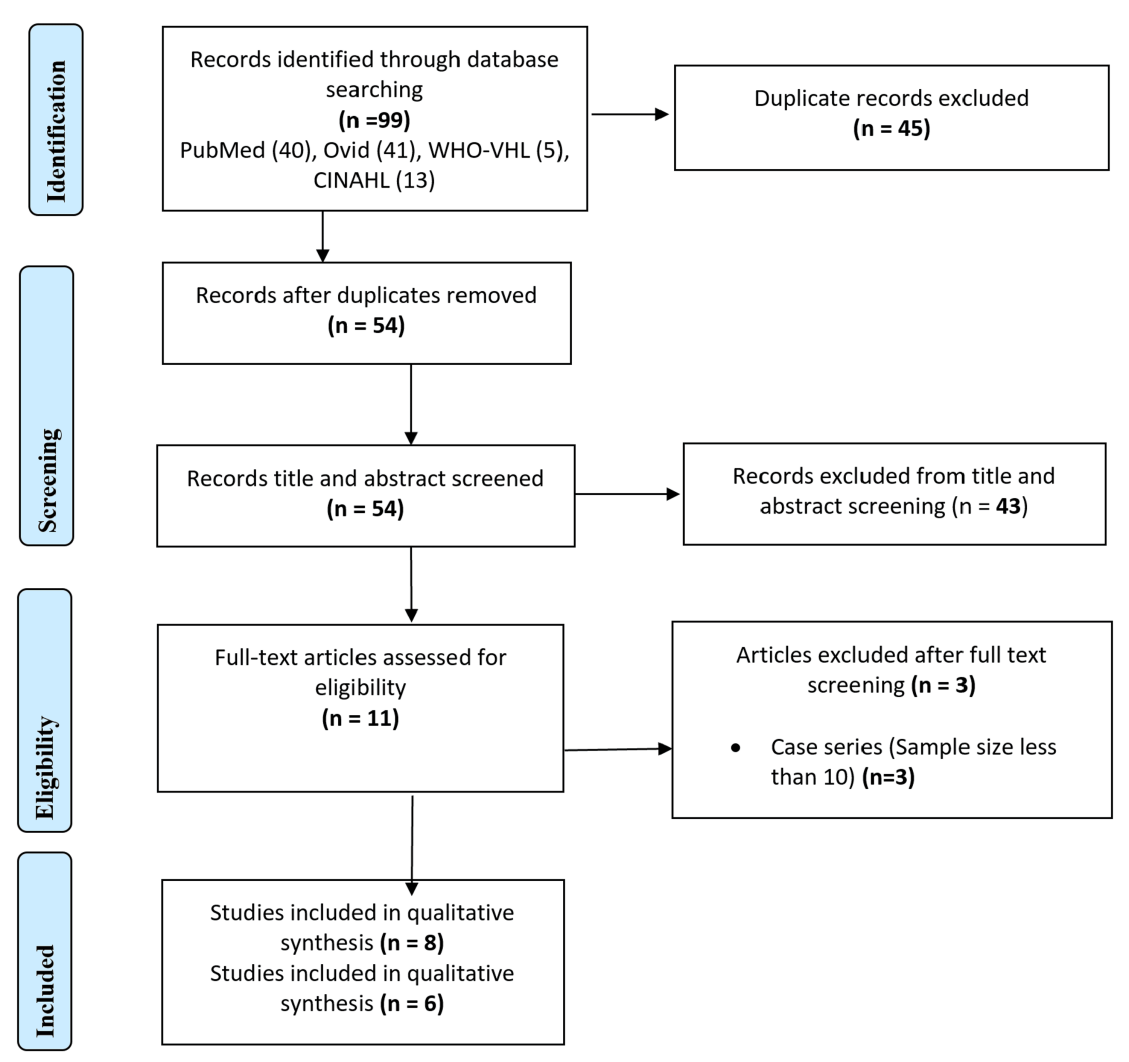
PRISMA flow chart for the study selection process Preferred Reporting Items for Systematic Reviews and Meta-Analyses

The schematic flow of the process employed for study identification and selection is presented in Figure 1. Initially, searching the electronic database yielded a total of 99 records. Following the removal of duplicates, 54 studies remained. These studies underwent a thorough title and abstract screening process. At this stage, 43 studies were excluded due to irrelevance. The full text of the remaining 11 records was screened, resulting in the exclusion of three studies, as they were review articles. Ultimately, a total of eight studies were selected for the systematic review [10-17].

Study Characteristics

The studies originated from six countries: the United States [11,15,17], Japan [13], Canada [16], Egypt [10], Poland [12], and Italy [14]. Study designs comprised cohort studies (n = 3) [11,14,17] and case series (n = 5) [10,12,13,15,16]. Collectively, these studies evaluated the use of 3D printing and customized implants in maxillofacial reconstruction, with a focus on surgical accuracy, time efficiency, cost, implant success rate, and long-term clinical outcomes (Tables 1, 2).

**Table 1:** Baseline characteristic findings for the included studies and the quality assessment

First Author/Year	Country	Study Design	Sample Size	Pathologies Treated	Quality Assessment
Moe et al. 2021 [11]	USA	Retrospective cohort	26	Squamous cell carcinoma, osteoradionecrosis, Ameloblastoma, and others.	7/11
Kanno et al. (2016) [13]	Japan	Prospective case series	20	Congenital, trauma, tumor-related facial bone deformities	9/10
Abo Sharkh & Makhoul (2020) [16]	Canada	Technical report and case series	19	Malignant and benign tumors, bony defects.	9/10
Khashaba et al. (2021) [10]	Egypt	Case series	10	palatal fistula, ameloblastoma, gunshot wound, mucormycosis.	8/10
Dowgierd et al. (2022) [12]	Poland	Prospective case series	21	Malignant/benign tumors, congenital deformities	8/10
Gugliotta et al. (2024) [14]	Italy	Retrospective cohort study	37	Tumors, trauma, congenital anomalies, osteomyelitis.	7/11
King et al. (2018) [17]	USA	Prospective comparative cohort study	38	Mandibular fractures	9/11
Williams et al. (2020) [15]	USA	Retrospective case series (comparison of lab vs in-house printing)	12	Benign and malignant tumors, trauma, osteoradionecrosis.	8/10

**Table 2:** Data extraction and the main outcomes from the included studies CAD: Computer-Aided Design; CAM: Computer-Aided Manufacturing; FDM: Fused Deposition Modeling; VSP: Virtual Surgical Planning; TMF: Temporalis Muscle Flap; PEEK: Polyether Ether Ketone.

Author	Type of Reconstruction	3D Printing Method	Graft Success Rate	Complication Reported	Accuracy Measures	Workflow Efficiency and the Cost
Moe et al. (2021) [11]	Fibula flap, scapula flap	In-house CAD/CAM using 3D printers	92.3%	Neck hematoma	The mean difference between planned and actual mandibular reconstructions was 1, with an RMS of 3.7290 m. excellent topographic accuracy.	In-house CAD/CAM for mandibular reconstruction using free flaps at a very low average cost of $3.87 USD per case.
Kanno et al. (2016) [13]	Custom-made CT-bone bone graft	3D inkjet printer	91%	Infection in graft sites	Compatibility in the shapes between the CT and the recipient bone was good during the surgery	Not reported
Abo Sharkh & Makhoul (2020) [16]	Fibula flap, scapula flap	FDM-based desktop 3D printer	Not reported as primary outcome	Not reported	Fit confirmed intraoperatively, no VSP inaccuracies reported	In-house VSP and 3D printing for bony reconstruction are both cost-effective and time-efficient, with a total cost of only $18.01 CAD per case and an average planning time of under 3 hours.
Khashaba et al. (2021) [10]	TMF	EOS P810 CAD/CAM machine	100%	Seroma, partial flap necrosis, transient facial nerve weakness	All patients indicated acceptance of the achieved results by the end of the follow-up period	Not reported as an outcome
Dowgierd et al. (2022) [12]	Free fibula flap, Iliac crest flap.	3D VSP with custom titanium plates via 3D printing	100%	Not reported	Mean deviation: 7.7 mm max, –6.09 mm min; not statistically significant. Indicating no discrepancy in preoperative and postoperative measurements	Not reported as an outcome
Gugliotta et al. (2024) [14]	Cranioplasty, zygoma, mandible, temporal fossa	PEEK implants via commercial CAD/CAM.	94%	Seroma, dehiscence infection.	Not quantified; high satisfaction and stability reported	Not reported as an outcome
King et al. (2018) [17]	Mandibular fracture fixation with or without 3D preadapted plates	On-site 3D model creation using FDM	Not reported as a primary outcome	Not reported	Not reported	Preoperative 3D printing and plate adaptation significantly reduced intraoperative plate adaptation time—from 22.8 minutes to 6.9 minutes—and cut the average surgical cost by over $1,600 per patient
Williams et al. (2020) [15]	Fibula free flap with immediate dental implant and prosthesis	In-house 3D printing.	93%	seromas.	Not quantified; clinical accuracy described as satisfactory	Not reported as an outcome

Patient Characteristics and Indications

The majority of the studies focused on adult populations, a total of 183 patients. Indications for reconstruction included oncologic resection (benign and malignant tumors), trauma, congenital deformities, osteoradionecrosis, and other causes. Reconstructed regions included the mandible, maxilla, zygomatic bone, temporal fossa, and cranial vault.

Reconstruction Types and 3D Printing Technologies

The most common reconstructive method was the fibula free flap, followed by iliac crest flaps, scapula flaps, and other reconstruction methods like temporal muscle flaps, as well as CT bone reconstruction flaps. Most of the studies used virtual surgical planning (VSP) integrated with CAD/CAM workflows to generate surgical guides, anatomic models, or definitive implants.

The 3D printing technologies that were employed across studies included in-house CAD/CAM using 3D printers, 3D inkjet printer models, fused deposition modeling (FDM)-based desktop 3D printers, the EOS P810 CAD/CAM machine, and 3D VSP with custom via 3D printing.

Workflow Efficiency and Cost Reduction

Among the eight included studies, two provided data regarding the cost reduction as well as the time reduction when using the 3D print technology. King et al. reported that preoperative 3D printing using the FDM significantly reduced intraoperative plate adaptation time from 22.8 minutes to 6.9 minutes and lowered the average surgical cost by over $1,600 per patient [17]. This highlights the clinical and economic value of incorporating preoperative 3D modeling and custom hardware preparation into surgical workflow studies. Similarly, Abo Sharkh et al. demonstrated that in-house VSP and 3D printing for bony reconstruction are both cost-effective and time-efficient, with a total cost of only $18.01 CAD per case and an average planning time of under three hours [16]. This further supports the practical value of in-house workflows in reducing both financial and time-related burdens in surgical preparation.

Accuracy and Radiographic Outcomes

Almost all the included studies reported either quantitative or qualitative assessments of implant accuracy and adaptation. Two studies provided quantification measures for accuracy and the adaptation of the implant between the preoperative and postoperative measurements. Dowgierd et al. (2022) assessed the accuracy of preoperative planning by comparing preoperative and postoperative measurements and reported a mean deviation ranging from -6.09 mm to 7.7 mm, which was not statistically significant. This indicates a high level of agreement between planned and achieved outcomes, supporting the reliability and adaptability of preoperative virtual planning and 3D-printed guides in clinical practice [12]. Additionally, Moe et al. (2021) reported a mean difference of just 1 mm between planned and actual mandibular reconstructions, with a root mean square (RMS) deviation of 3.73 mm, indicating excellent topographic accuracy [11]. These results highlight the precision achievable through preoperative virtual planning and patient-specific 3D-printed reconstruction techniques. The remaining studies confirmed excellent intraoperative fit, patient satisfaction, and postoperative CT imaging.

Clinical Outcomes

The most common clinical outcomes that were discussed in most of the studies were the implant success rate and the complications encountered. Implant or graft success rates were consistently high. Six studies reported specific implant integration rates ranging from 91% to 100% [10-15]. On the other hand, the complication rate was not significant for most of the study, with the seroma being the most common, followed by minor infection at the site of implants.

Meta-Analysis

**Figure 2:**
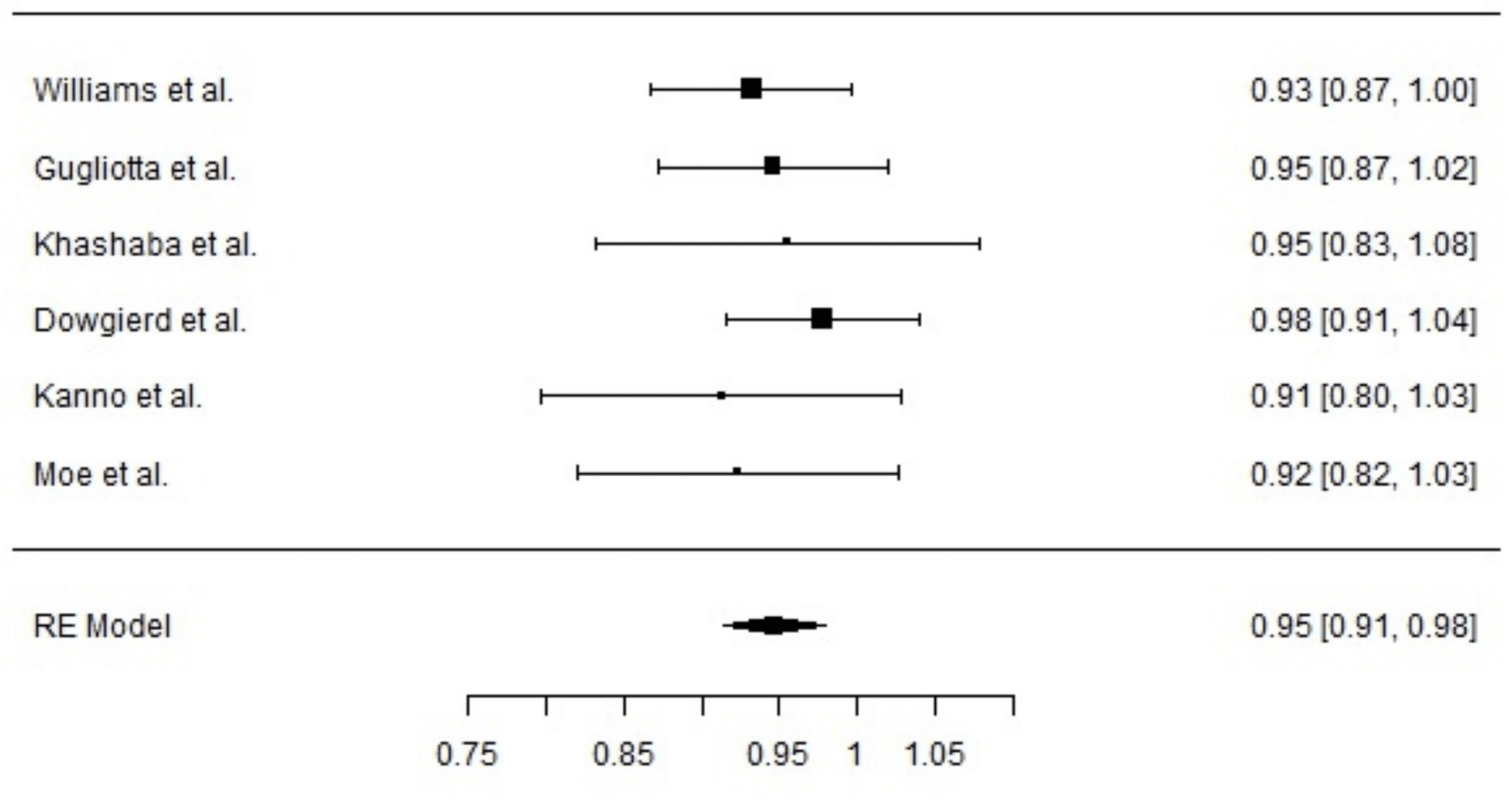
The forest plot demonstrating the high success proportions across the studies Williams et al. [15], Gugliotta et al. [14], Khashaba et al. [10], Dowgierd et al. [12], Kanno et al. [13], Moe et al. [11].

Six studies met the eligibility criteria and were included in the meta-analysis, meaning that only studies that reported implant success rate as one of the outcomes were included. The pooled estimate for implant success was 94.7% (95% CI: 91.4% to 98.0%), as calculated using a random-effects model. The result was statistically significant (Z = 56.5, p < 0.001) (Figure 2).

Heterogeneity

**Figure 3:**
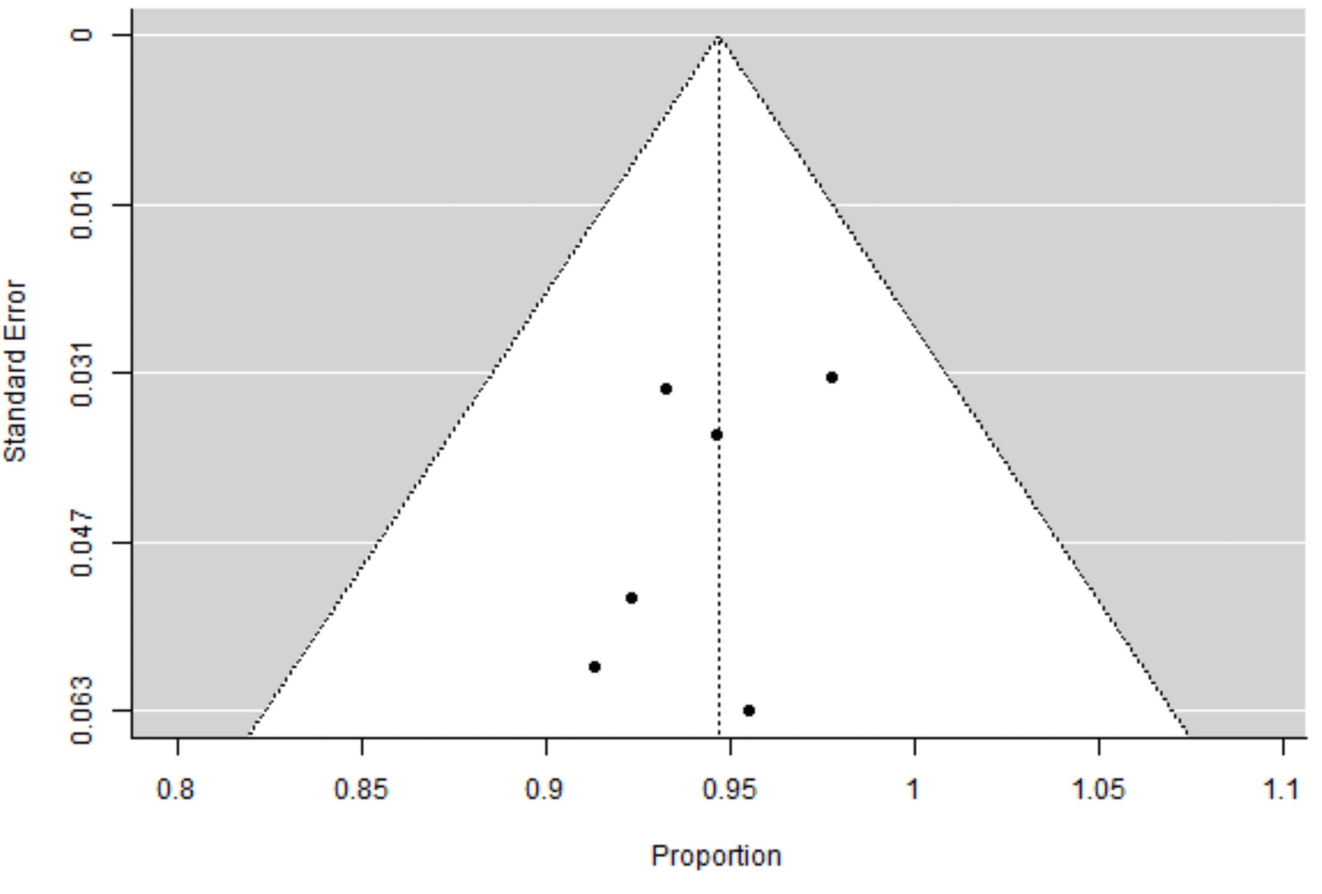
The funnel plot showing symmetric distribution around the pooled effect size

There was no significant heterogeneity among the studies (Q = 1.671, df = 5, p = 0.893), with I² = 0%, indicating consistent results across the included studies. The between-study variance (τ²) was effectively zero, confirming homogeneity of effect sizes. These findings support the consistency and reliability of implant outcomes across varied clinical settings and surgical teams. The fail-safe N analysis estimated that 6541 missing studies with null results would be needed to reduce the overall significance to a non-significant level (p > 0.05), supporting the robustness of the findings. Based on both the Kendall’s rank correlation test (Tau = -0.200, p = 0.719) and the Egger’s regression test (Z = -0.613, p = 0.540), there is no evidence of funnel plot asymmetry or publication bias (Figure 3).

Discussion

This systematic review and meta-analysis provide compelling evidence supporting the clinical efficacy and logistical advantages of using 3D-printed PSIs in maxillofacial reconstruction. The pooled success rate of 94.7% demonstrates a high degree of reliability and integration success across diverse patient populations and surgical contexts, corroborating the growing adoption of PSIs as a viable alternative to traditional reconstructive approaches.

The absence of significant heterogeneity (I² = 0%) in our meta-analysis underscores the consistency of outcomes across the included studies, despite variations in anatomical sites, materials used, and surgical teams. This consistency may reflect the increasingly standardized application of VSP and CAD/CAM technologies, which allow for meticulous preoperative design and improved intraoperative accuracy. These technologies, as evidenced in studies such as those by Schulze et al. (2024) and Moellmann et al. (2022), contribute to the high precision of implant fit, often within sub-millimetric tolerances, which is critical in anatomically complex regions like the mandible and midface [5,6].

The economic feasibility of PSIs was another key theme. In-house printing, reported in several included studies, dramatically reduced production costs and turnaround times, sometimes from weeks to under 24 hours. This democratization of access to PSIs is especially significant for resource-constrained settings, where reliance on external fabrication services can be both financially and logistically prohibitive. The cost-effective findings align with previous reports by Du et al. (2023), reinforcing the notion that 3D printing workflows can be scaled and localized without compromising clinical outcomes [2].

From a complication standpoint, the included studies report relatively low rates of adverse events. The most common complications were seroma and minor infections, which were generally self-limited or manageable without surgical revision. Importantly, there were no reported instances of catastrophic implant failure or misfit requiring major reintervention. These outcomes compare favorably with those of conventional graft-based methods, which carry risks of donor-site morbidity, graft resorption, or contour loss over time.

Nonetheless, several limitations merit discussion. First, while our meta-analysis included six high-quality studies, the overall pool of available literature remains limited, and most included studies were observational in nature. The lack of RCTs reduces the strength of evidence and may introduce selection bias. Moreover, heterogeneity in reporting standards (e.g., outcome measures, radiographic validation, and follow-up periods) complicates direct comparisons across studies. Future research should prioritize multicenter trials with uniform reporting frameworks and long-term follow-up to evaluate implant durability, functional outcomes, and patient satisfaction.

A second limitation is the diversity of materials used ranging from titanium and PEEK to calcium phosphate-based biomaterials which may influence osseointegration, mechanical behavior, and biocompatibility. While titanium remains the most used and well-validated material, future studies should aim to delineate material-specific performance differences and their impact on complication rates or imaging follow-up.

Finally, regulatory and ethical considerations regarding PSI manufacturing, quality assurance, and intraoperative decision-making are still evolving. The integration of engineers and radiologists into surgical planning teams has improved design precision but also necessitates new training paradigms and interdisciplinary workflows, as noted by Memon et al. (2022). Formal guidelines for PSI production and validation like those used in pharmaceutical or medical device development are needed to ensure safety and reproducibility [1].

## Conclusions

This systematic review and meta-analysis underscore the clinical utility and operational advantages of 3D-printed PSIs in maxillofacial reconstruction. With a pooled success rate nearing 95% and minimal heterogeneity across studies, PSIs demonstrate excellent integration, accuracy, and safety. Moreover, the adoption of in-house 3D printing workflows offers significant reductions in production time and cost, broadening access in both high-resource and resource-limited settings.

While the body of evidence is growing, the current literature is predominantly composed of observational studies with short-to-intermediate follow-up durations. Future research should prioritize standardized outcome reporting, long-term durability assessments, material comparisons, and RCTs to further validate these promising results and support the development of clinical guidelines for PSI use in craniofacial surgery.
